# Programme for the Galveston Meeting

**Published:** 1888-04

**Authors:** 


### PROGRAMME FOR THE GALVESTON MEETING.

TEXAS STATE MEDICAL ASSOCIATION, APRIL, 24.

A note from Dr. J. F. Y. Paine, chairman of the committee of arrangements, conveys the following interesting information:

The committee of arrangements are laboring assiduously to make the coming convention a success, to which end, a number of innovations will be introduced into the programme, looking toward a higher order, and wider scope of work.

As already provided, the first day of the session will be devoted to the consideration of amendments of the constitution and by-laws. The two following days will—as far as practicable—be wholly given up to Section work. The assembly will be divided into four Sections, provided with distinct apartments, and all in progress at the same time. This scheme will afford opportunity for all worthy papers to be read and discussed. There will be a general meeting each morning during the session, at which Chairmen of Sections will deliver addresses, and reports of the Judicial Council be received and upon acted, when adjournment will take place to the respective Sections. An executive session will be held on Friday, the last day of the sitting, for the transaction of miscellaneous business.

